# Measuring the ambiguity tolerance of medical students: a cross-sectional study from the first to sixth academic years

**DOI:** 10.1186/1471-2296-15-6

**Published:** 2014-01-09

**Authors:** Anne Weissenstein, Sandra Ligges, Britta Brouwer, Bernhard Marschall, Hendrik Friederichs

**Affiliations:** 1 Department of Gastroenterology and Hepatology, University Hospital of Cologne, Kerpener Strasse 62, Cologne, 50937 Germany; 2 Institute of Biostatistics and Clinical Research, University of Muenster, Schmeddingstraße 56, Muenster, 48149 Germany; 3 Institute of Student affairs and Medical Education - IfAS, University of Muenster, Studienhospital, Malmedyweg 17-19, Muenster, 48149 Germany; 4 Institute of Student affairs and Medical Education - IfAS, University of Muenster, Albert - Schweitzer - Campus 1/Gebäude A6, Muenster, 48149 Germany

**Keywords:** Ambiguity tolerance, Medical students, General practitioners

## Abstract

**Background:**

Tolerance of ambiguity, or the extent to which ambiguous situations are perceived as desirable, is an important component of the attitudes and behaviors of medical students. However, few studies have compared this trait across the years of medical school. General practitioners are considered to have a higher ambiguity tolerance than specialists. We compared ambiguity tolerance between general practitioners and medical students.

**Methods:**

We designed a cross-sectional study to evaluate the ambiguity tolerance of 622 medical students in the first to sixth academic years. We compared this with the ambiguity tolerance of 30 general practitioners. We used the inventory for measuring ambiguity tolerance (IMA) developed by Reis (1997), which includes three measures of ambiguity tolerance: openness to new experiences, social conflicts, and perception of insoluble problems.

**Results:**

We obtained a total of 564 complete data sets (return rate 90.1%) from medical students and 29 questionnaires (return rate 96.7%) from general practitioners. In relation to the reference groups defined by Reis (1997), medical students had poor ambiguity tolerance on all three scales. No differences were found between those in the first and the sixth academic years, although we did observe gender-specific differences in ambiguity tolerance. We found no differences in ambiguity tolerance between general practitioners and medical students.

**Conclusions:**

The ambiguity tolerance of the students that we assessed was below average, and appeared to be stable throughout the course of their studies. In contrast to our expectations, the general practitioners did not have a higher level of ambiguity tolerance than the students did.

## Background

Budner (1962) defines intolerance of ambiguity as “the tendency to perceive ambiguous situations as sources of threat” and tolerance of ambiguity as the tendency to “perceive ambiguous situations as desirable” [1]. Ambiguity is a type of risk, in that the probability of the outcome is unknown [2]. Ambiguous situations cannot be adequately structured or categorized by the individual because of insufficient cues [1]. Tolerance of ambiguity has been associated with several positive traits, such as originality and openness to new ideas [3]. In contrast, intolerance of ambiguity has been associated with lower mental flexibility as well as other negative personality traits, such as mental rigidity, conformity, and ethnic prejudice [1,3,4]. The framework of our study is the cognitive psychological conceptualization of ambiguity tolerance. The correlates of AT in this framework are risk taking propensity and uncertainty orientation-any trait orientation toward stimuli that involve risk, uncertainty, complexity, unfamiliarity, and related perceptions.

Employment in the health care industry is characterized by novelty, complexity, and sometimes insolubility [5]. Thus, physicians may encounter very complex situations, as they tend to patients whose treatments and diagnoses reflect a wide continuum of ambiguity. As Geller (2013) summarized, physicians who have a low tolerance of ambiguity are more likely to recall mammograms [6], increase patient charges [7], withhold negative genetic test results [8], fear malpractice litigation, and thus engage in defensive practice [9], experience discomfort in the context of death and grief [10], exhibit greater test-ordering tendencies, and demonstrate failure to comply with evidence-based guidelines [11].

Tolerance for ambiguity also plays an important role on the attitudes and behaviors of medical students. A considerable body of literature exists regarding the tolerance level of ambiguity of medical students. Consequentially, the following traits have been associated with a low tolerance of ambiguity in this population: negative attitudes toward the underserved [12,13] and fear of making mistakes [14]. Conversely, higher tolerance of ambiguity has been associated with greater leadership abilities in medical students [15] as well as increased willingness to practice in rural areas [16,17]. It is possible that the way students deal with ambiguity is malleable [18]. Geller (2013) has attempted to explain why: medical students with a high tolerance of ambiguity entering medical school are drawn to uncertainties characterized by medicine and thus have the opportunity to further develop their ambiguity - related communication and decision-making skills. These students would then have the opportunity to further develop their ambiguity-related communication and decision-making skills. The result is a positive feedback loop in which the tolerance of ambiguity increases in these students [5]. In a similar manner, a negative feedback loop may operate for students with a low ambiguity tolerance, as they may tend to avoid ambiguous situations and thus become even less tolerant [5]. By assessing and evaluating the tolerance of ambiguity among medical students, it may be possible to determine whether this trait is stable, and whether tolerance can be taught and/or developed. Although several studies have compared ambiguity levels across several cohorts of students, a thorough and systematic literature review failed to uncover a study in which tolerance of ambiguity was compared across all years of medical school.

To address this issue, we conducted a study to evaluate the ambiguity tolerance of students from the first to the sixth academic years. We also compared ambiguity tolerance between students and general practitioners. We chose to assess ambiguity tolerance in general practitioners as this population is thought to have a higher ambiguity tolerance than specialists, because of their limited access to sophisticated diagnostic equipment, lack of opportunities to consult specialists, and unselected patients with a broad range of medical concerns [16,19].

## Methods

### Study design and participants

Our study was conducted in the summer semester of 2013 at the medical school of the Westphalian Wilhelms University in Muenster, Germany. We used a cross-sectional design to evaluate ambiguity tolerance in 622 medical students from the first to the sixth academic years, as well as 30 general practitioners. In Germany, medical school is completed in six years, and is divided into preclinical (first two years) and clinical (last four years) sections. In the last year, or ‘practical’ year, students rotate through various hospital departments. We recruited students by approaching them in the context of their annual progress test, which is used to assess cumulative increases in medical knowledge. We invited exactly half of the students from each year to participate in our study. The general practitioners that we surveyed were members of the medical teaching staff at the medical school. They were approached during a voluntary meeting, which occurs once a semester. We used the standard alpha-level of 0.05 for significance and a power level of 0.8. Thus, we needed at least 28 participants to detect a large effect size (r = 0.5) [20].

We obtained informed consent from all participants prior to the study. As determined by the Ethics Committee of the Chamber of Physicians at Westphalen-Lippe and the Medical School of Westphalian Wilhelms University in Muenster, no ethical approval procedure was necessary.

### Outcome measures and measuring instrument

Measurements were conducted using the Inventory for measuring ambiguity tolerance (IMA) by Reis (1997, Additional file 1) [21]. The IMA comprises 40 items, divided into the following five areas:

Ambiguity tolerance with respect to:

● apparently insoluble problems (PR)

● social conflicts (SC)

● parental image (PI)

● role stereotypes (RS)

● openness to new experiences (OE).

As our aim was to conduct a survey in the context of an academic profession, we shortened the sections of the IMA that addressed parental image and role stereotypes. For the remaining three areas, the Cronbach’s alpha values ranged from .78 to .86, indicating acceptable levels of reliability.

The students answered 20 questions on a 6-point Likert scale with the following anchors: 1-“strongly agree”, 2-“agree”, 3-“somewhat agree”, 4-“somewhat disagree”, 5-“disagree”, and 6-“strongly disagree”. Ambiguity tolerance was assessed for the three scales regarding openness to new experiences (OE, eight questions in total), social conflicts (SC, six questions in total), and apparently insoluble problems (PR, six questions in total). Age and gender-specific norms have been documented for each scale, resulting in a classification system, developed by Reis (1997), that defines standard ranges in terms of percentage (e.g. 1–10%, 11–20%, up to 91–99%). For all such scales, a high percentage indicated a high manifestation of a measured attribute and a low percentage indicated a low manifestation of a measured attribute. We also collected demographic characteristics such as age, gender, and academic year.

### Data analysis

Acquired data were entered and analyzed using the statistical software IBM Statistics SPSS 21 and R version 3.0.1 (R Core Team, 2013). We used an unpaired two-sided *t*-test to assess the difference between male and female students as well as the difference between medical students and general practitioners. We used an F-test to assess the differences between students in different academic years (we conducted each test separately to obtain a score for OE, SC, and PR). Prior to our analysis, we checked for normal distribution and homoscedasticity. The local significance level was set to 0.05 for each test. No adjustment for multiple testing was performed.

## Results

In total, we obtained 564 complete data sets (return rate 90.1%) from a total of 622 medical students. We obtained 29 questionnaires (return rate 96.7%) from general practitioners. In terms of student cohort (first to sixth year), we obtained 145 (97.3%) data sets from first-year students, 117 (84.1%) data sets from second-year students, 107 (84.9%) data sets from third-year students, 78 (86.7%) data sets from fourth-year students, 97 (98.9%) data sets from fifth-year students, and 20 (71.4%) data sets from sixth-year students.

The mean age (standard deviation) of the medical students was 23.2 (3.8) years, and 61.5% of the students were female. The mean age of the general practitioners was 51.3 (8.6) years, and 20.7% were female. The characteristics of our student respondents were comparable to the characteristics of the general student population, as ~50–60% of all medical students in Germany are female (based on statistics from 2000) [22]. However, the percentage of female respondents in our group of general practitioners was low with respect to the overall demographic in Germany, where about 41.3% of all GPs are female [23].

On average, participants obtained a mean score (95% confidence interval) of 21.9 (21.5–22.3) for OE, 23.8 (23.8–24.2) for SC, and 16 (15.7–16.4) for PR. With respect to the reference groups defined by Reis, the average for medical students was in the 1–10% band regarding their ambiguity tolerance for OE and PR and in the 61–70% band regarding their ambiguity tolerance for SC. We detected a significant increase in PR score between the first (mean score 15.1 points) and the second (mean score 16.5 points, p = .007) academic years, as well as between the fourth (mean score 16.7, p = .011) and fifth academic years (mean score 16.6, p = .009). OE and SC scores also significantly increased between the first (OE: 21.2, SC: 23.2) and second academic years (OE: 22.2, p = .026; SC: 25.1, p = .002). However, these differences were not indicative of a larger trend, as the students remained in their respective percentage band.

**Figure 1:**
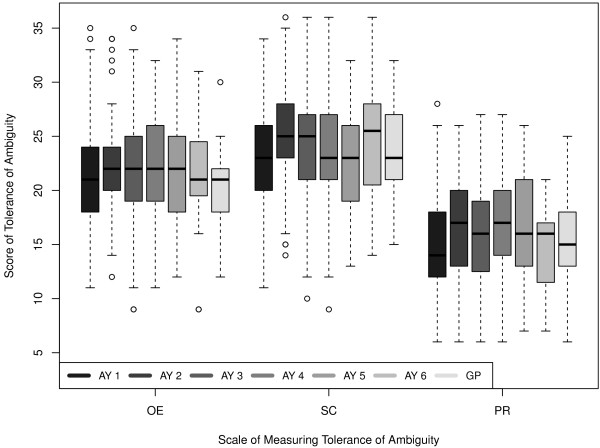
Ambiguity tolerance of general practitioners and medical students in the first to the sixth academic years.

Figure 1 shows the scores for each of the three scales with respect to academic year together with the results from the general practitioners.

In terms of openness to new experiences, male medical students were more tolerant (mean OE score of 22.6, 1-10% band) than female students (mean OE score of 21.4, 1–10% band, p = .002). Regarding social conflicts, female students were more tolerant (mean SC score of 24.1, 71–80% band) than male students (mean SC score of 23.2, 61–70% band, p = .029). Finally, female students exhibited greater ambiguity tolerance for insoluble problems than male students (mean PR score of 16.2, 1–10% band, compared with a mean PR score of 15.7, 1–10% band, p = .179). The differences that we observed were modest, although they reached a significant level.

We found no significant differences between the scores (95% confidence interval) obtained by the general practitioners (OE score 20.5 (19.2–21.7), SC score 23.6 (21.8–25.3), PR score 15.3 (13.4–17.2)), and those obtained by the medical students (p = .111 for OE score, p = .8 for SC score and p = .404 for PR score).

## Discussion

The results of our study indicate that medical students have a very low level of ambiguity tolerance regarding openness to new experiences and in their approach to apparently insoluble problems. Here, we detected gender-specific differences with respect to openness to new experiences and social conflicts. However, the gender-based differences in openness to new experiences were minimal (the average for both female and male students was in the 1–10% band). The observed differences in approach to social conflicts (average for female students was in the 71–80% band and the average for male students was in the 61–70% band) may have been due to a behavioral response bias regarding social desirability. Although we observed differences between the various academic years, these differences were not significant with respect to the reference groups suggested by Reis (1997). Although general practitioners are thought to possess a higher level of ambiguity tolerance, we did not detect any clinically relevant dissimilarities between our sample and the reference groups suggested by Reis (1997).

In contrast with DeForge (1989) and Tatzel (1980), who found younger students to be more intolerant of ambiguity than older students [3,24], we did not detect cohort-specific differences. Corroborating the findings of Reis (1997), who stated that ambiguity tolerance tends to remain stable from adolescence to middle age, and does not change until around age 50 [21], we found no differences in ambiguity tolerance between the academic years of medical school. In general, our student population appeared to be more intolerant of ambiguity than those studied by Reis [21]. Our findings may indicate that medical students have changed in terms of their ambiguity tolerance in the years since Reis’ study. Despite the trend described by Fox [25], who perceived students in the 1970s as being more capable of dealing with uncertainty than those in the 1950s, DeForge hypothesized that, owing to their increasing dependence on technology, students today may be seeking more structure than their predecessors, and thus may perceive ambiguity as more of a threat [24]. As medical students who are more tolerant of ambiguity tend to choose more unstructured specialties, such as family practice [1,26], we expected that general practitioners would have a higher ambiguity tolerance than students. However, like DeForge, who found no differences in ambiguity intolerance based on the medical specialization preferences of incoming medical students, we found no clinically relevant differences between general practitioners and medical students [24,27]. If general practitioners do exhibit a different level of ambiguity tolerance to medical students, this may be an important educational consideration, alongside the efforts to train students to have sound diagnostic skills, in which accuracy and attention to detail play a central role in academic and professional success [28].

There are several limitations to our study. First, the study design is not longitudinal, thus it is not possible to make conclusions about the development of ambiguity tolerance in individual students. Additionally, the generalizability of our study may be limited, as we only questioned students from one school.

## Conclusions

In conclusion, the ambiguity tolerance of our students was clearly below average. Ambiguity tolerance is critical, as evidence suggests that if medical students possess a high tolerance for ambiguity, they may provide a higher quality of care in ambiguous conditions. In addition, they might demonstrate increased humility, which is necessary for moral character formation in terms of one’s role in the medical practice [5]. We are optimistic, as we found a relatively high level of ambiguity tolerance towards social conflicts, meaning that the medical students in our study may be interested in exchanging views about sensitive and controversial issues. We found no differences in ambiguity tolerance between students in the first and sixth years of medical school. However, it is important to find answers to the question whether or not ambiguity tolerance is malleable, and can thus be taught or developed or if ambiguity tolerance is determinate, and thus may be useful as a selection tool in medical education. A longitudinal study is necessary to further explore this issue.

## Competing interests

The authors declare that they have no competing interests.

## Authors’ contributions

HF conceived of the study, participated in its design and coordination, and helped to draft the manuscript. BB participated in the data acquisition and data analysis. BM participated in the study design and coordination. SL participated in the data analysis and helped draft the manuscript. AW participated in the data analysis, helped with the study coordination, and helped draft the manuscript. All authors read and approved the final manuscript.

## Authors’ information

AW has a medical degree and works in the department of gastroenterology and hepatology at the University hospital of Cologne. SL has a doctoral degree and works as a statistician at the Institute of Biostatistics and Clinical Research in Muenster. BB works as a research assistant at the simulation center in Muenster. BM is the dean of the medical faculty and the head of the Institute of medical education and student affairs at the University of Muenster. HF is the head of the simulation center at the medical school in Muenster and has a master’s degree in Medical Education (MME).

## Pre-publication history

The pre-publication history for this paper can be accessed here:

http://www.biomedcentral.com/1471-2296/15/6/prepub

## Supplementary Material

Additional file 1 Questionnaire based on the ambiguity tolerance inventory by Reis, courtesy of Asanger Verlag, GmbH. Click here for file
