# Supplementary material for: Measuring the ambiguity tolerance of medical students: a cross-sectional study from the first to sixth academic years
Source: BMC Fam Pract. 2014 Jan 9;15:6. doi: 10.1186/1471-2296-15-6 (PMC3897997; doi:10.1186/1471-2296-15-6)
Supplement: Additional file 1 — Questionnaire based on the ambiguity tolerance inventory by Reis, courtesy of Asanger Verlag, GmbH. [file 1471-2296-15-6-S1.pdf]

Westfälische Wilhelms-Universität Münster

IfAS Institut für Ausbildung und Studienangelegenheiten

Inventory for Measuring Ambiguity tolerance (IMA):

Ambiguity tolerance is the tendency to evaluate contradictions, inconsistencies, and ambiguous information in terms of their complexity and regard them in a positive manner (J. Reis). Medicine is known as "the science of unsecured, art of probability" (W. Osler); thus, personal levels of ambiguity tolerance may impact success in the medical profession.

Please provide answers to the questions below and enter your estimates in the provided spaces.
We would like to use the anonymous test results for a study dealing with ambiguity tolerance in medical students in Germany. Study participation is completely voluntary. The collected data will only be used for this purpose. Under no circumstances will the data be distributed to unauthorized third parties, and after evaluation the data will be destroyed.

How old are you? _________ *years*

Gender ? ❒ *male* ❒ *female*

In which semester (S) are you?
❒ *1./2. S* ❒ *3./4. S* ❒ *5./6. S* ❒ *7./8. S* ❒ *9./10. S* ❒ *Practical Year*

	Strongly agree	Agree	Partly agree	Partly disagree	Disagree	Strongly disagree
I like to plan my vacations in advance.	❍	❍	❍	❍	❍	❍
Problems that appear to be insoluble feel like personal challenges to me.	❍	❍	❍	❍	❍	❍
I enjoy going to parties where I can meet new people.	❍	❍	❍	❍	❍	❍
I do not like to deal with problems that appear to have no solution.	❍	❍	❍	❍	❍	❍
Sometimes it is fun to do new things with my friends.	❍	❍	❍	❍	❍	❍
Unless I was receiving a lot of money, I wouldn‘t choose to spend my time on seemingly insoluble problems.	❍	❍	❍	❍	❍	❍
I try to avoid disputes.	❍	❍	❍	❍	❍	❍
I try to avoid people who like to argue.	❍	❍	❍	❍	❍	❍
I need a familiar environment to feel comfortable.	❍	❍	❍	❍	❍	❍
I like to visit countries where I have not previously travelled.	❍	❍	❍	❍	❍	❍
I try to avoid confrontation.	❍	❍	❍	❍	❍	❍
I don’t like surprises.	❍	❍	❍	❍	❍	❍
I try to avoid seemingly insoluble problems.	❍	❍	❍	❍	❍	❍
I try to get along with everyone.	❍	❍	❍	❍	❍	❍
It is important to me that others do not think of me as belligerent.	❍	❍	❍	❍	❍	❍
It seems pointless to me to concern myself with problems that seem insoluble.	❍	❍	❍	❍	❍	❍
I like to talk to friends about innocuous topics.	❍	❍	❍	❍	❍	❍
I am interested in foreign cultures.	❍	❍	❍	❍	❍	❍
Dealing with seemingly insoluble problems can be useful, even if I don’t solve them.	❍	❍	❍	❍	❍	❍
I am easily excited by new hobbies.	❍	❍	❍	❍	❍	❍

Thank you for your participation !
